# Mediastinal mature cystic teratoma in a child: A case report study^☆^

**DOI:** 10.1016/j.ijscr.2023.107904

**Published:** 2023-01-13

**Authors:** Ayham Ghareeb, Fawaz Al Sharif, Ayham Alyousbashi, Methad Dawarah, Amjad Ghareeb, Husam Dalati

**Affiliations:** a Faculty of Medicine, Damascus University, Damascus, Syria; b Pediatric Surgery Department, Children's University Hospital, Damascus University, Damascus, Syria

**Keywords:** Case report, Mature teratoma, Hydatid cyst, Lung abscess, Mediastinal tumors, Pediatric

## Abstract

**Introduction:**

A mediastinal teratoma is a very rare disease, in which a relatively large mass grows in the space between the two lungs. Although it is a congenital abnormality, it is diagnosed late in the second or third decade. It could be benign or malignant, symptomatic or asymptomatic.

**Presentation of case:**

A five-year-old child presented with an acute respiratory distress episode due to an acute infection, unresponsive to first-line antibiotics and had a history of similar recurrent pulmonary infections. On imaging, the patient showed an abnormal mediastinal mass which was speculated to be a hydatid cyst or abscess; however, it was later diagnosed during surgery as a mature mediastinal cystic teratoma.

**Discussion:**

Mediastinal tumors are slow-growing tumors that are mostly benign and asymptomatic, but sometimes they may grow, reaching a state where they can affect the neighboring anatomical structures, causing symptoms, which may lead to misdiagnosis.

**Conclusion:**

Teratomas, as a differential diagnosis, should be considered in children with recurrent pulmonary infection and radiological findings that resemble masses in the chest. This emphasizes on the importance of performing detailed radiological and lab investigations to set an appropriate diagnosis to ensure a better treatment and prognosis.

## Highlights

• Mediastinal mature cystic teratoma in a child
• Misdiagnosis is common in mediastinal teratoma, such as: hydatid cyst and lung absence.
• Mediastinal teratoma must be considered as a differential diagnosis in mediastinal lesions.
• The necessity of doing a CT in multiple recurrent and unresponsive pneumonia

## 1 Introduction

Mediastinal teratomas originate from the pluripotent embryonic cells which are going to differentiate later into tissues with all three germ cell layers. These teratomas have an equal distribution in both genders, and mostly get diagnosed at the second and third decades of life [1]. The incidence of teratomas is approximately 1 in 4000 live births worldwide. The most common sites of representations are 1. sacrococcygeal (40 %), 2. ovarian (25 %), 3. testicular (12 %), 4. cerebral (5 %), and other places, including neck and mediastinum (18 %) [2]. The frequency of mediastinal teratomas ranges from 1 % to 5 % [3]. However, pure mediastinal teratomas are often found in the first 5 years of children's life, and malignant variants of teratomas are more common in adolescence [4], [5]. They can be classified into mature solid or cystic teratoma, immature teratoma, and teratoma with malignant transformation [6]. This case presents a mediastinal mature cystic teratoma in a five-year-old child who came with recurrent pulmonary infections and was thought to be a mediastinal hydatid cyst or an abscess. This case report is compliant with the SCARE Guidelines [7], which helped to improve the transparency and quality of this case report.

## 2 Case report

A five-year-old female child admitted to the hospital due to fever (40 °C), dyspnea and chest pain spreading to the right shoulder and arm. The patient had history of recurrent pneumonia since the age of 6 months, which was always treated using antibiotics. Heart rate, blood pressure and oxygen saturation were within normal range. Clinical examination showed diminished respiratory sounds in the right lung field, mainly at the base, with dullness on percussion with increased resonance.

Lab results showed increased WBCs count (22,100*10^9^/L / N=74% / L=21%), platelets count (764,000*10^9^/L), and hemoglobin was decreased (10.4 mg/dl). Electrolytes were within normal range. Chest X-ray (CXR) showed diffused opacification in the middle and lower right lung field. A computed tomography (CT) scan showed alveolar opacification within the right middle pulmonary lobe, implying the presence of lobar pneumonia in addition to condensation between the previous opacification and the heart, implying the presence of an idiopathic cyst.

**Fig. 1:**
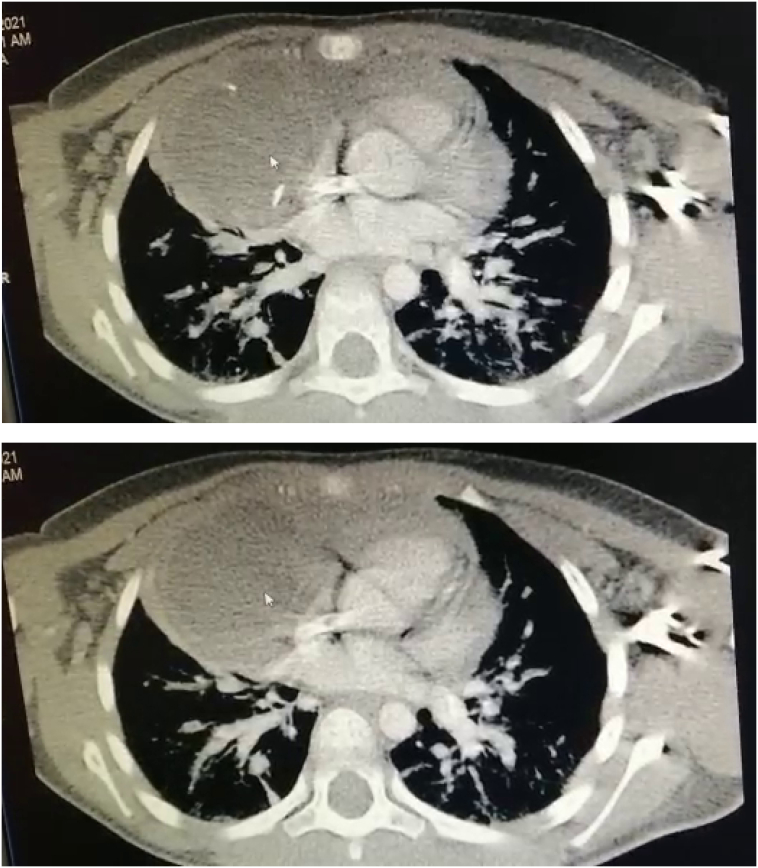
The first and second CT scan with contrast, showing the position of the mass and the neighboring anatomical structures.

The child was given antibiotics, but with no improvement. Then the patient was put on (vancomycin, ceftriaxone, amikacin). Clinical improvement was noticed in the following days with no radiological improvement on the CXR. Therefore, a contrast CT scan was repeated which showed a 50 mm diameter cyst near the heart, with calcified walls; regressed alveolar infiltrates were also noticed in comparison to the previous CT (Fig. 1).

Based on the current findings, the child was transferred to the “Children's University Hospital” so that the mass can be further studied. The child regiment was changed again to (gentamicin and amoxicillin) upon admission. Based on the radiological findings, the diagnosis of Hydatid cyst or lung abscess was considered most likely as they are common in eastern Syria. However, serum antibodies (Echinococcus Ab 1H) were ordered for hydatid cysts which yielded negative results.

Nevertheless, a surgical approach was deemed appropriate. The procedure was done through an anterior lateral incision on the fifth intercostal space. A mass was found filling the mediastinum in between the heart and the aorta, with adhesions to the pericardium but no adhesions to the lung. The mass compressed the left lung entrance, with no noticeable effects on the heart.

**Fig. 2:**
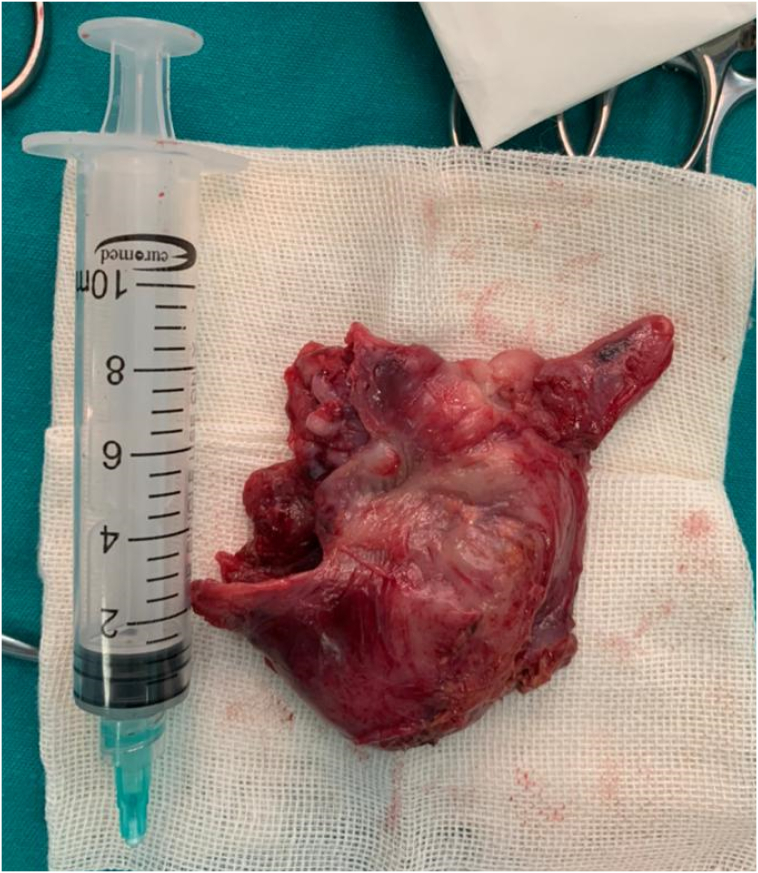
The mass after excision.

During the procedure, the mass was treated as if it was an abscess, and during the drainage, a clear viscous liquid was extracted from it. The mass was measured (5.5 ∗ 5.5 ∗ 2.5 cm) and connected to another triangular mass measured (3 ∗ 2 ∗ 1 cm), and it was completely removed after releasing the adhesions (Fig. 2).

**Fig. 3:**
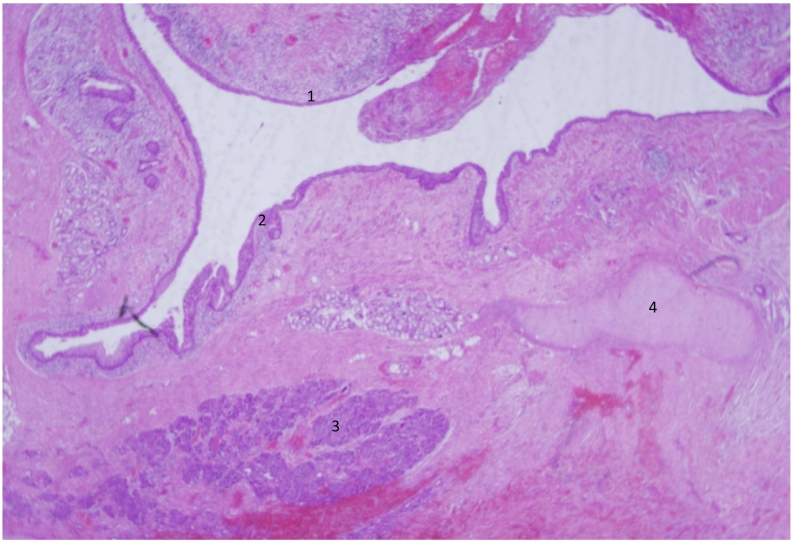
A histological overview from the mass showing: 1. Pulmonary epithelial, 2. prickle cell layer, 3. pancreatic tissues, 4. connective tissues with chondrocytes.

Pathologically, a presence of different sized cystic formation was found, with varying epithelial layers (Malpighian layer, columnar layer), mucous glands, pancreatic islets, connective tissues with chondrocytes and adipose, cartilage and osseous tissue with granuloma (Figs. 3 + 4).

**Fig. 4:**
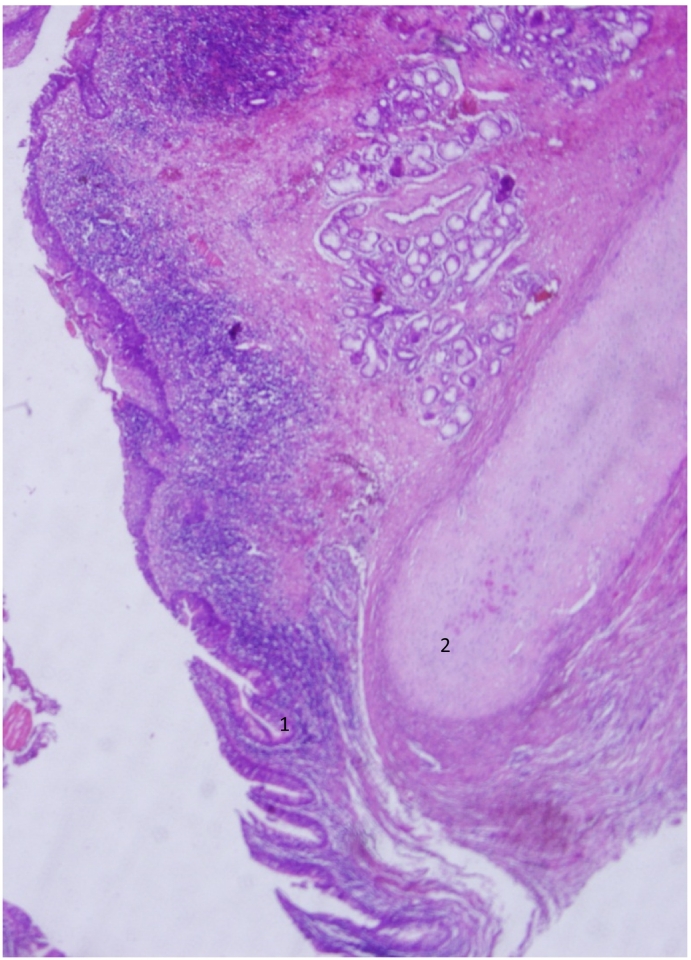
A histological overview of the mass showing: 1. prickle cell layer, 2. connective tissues with chondrocytes.

Based on the pathological findings the diagnosis was made as mature cystic teratoma with a secondary infection.

Due to low resources, it was decided to do an αFP test only after the surgery, which returned a value within the normal range (1.4 IU/ml), supporting the pathological diagnosis and ensuring that the cystic teratoma is mature and has no malignant aspect.

The thorax drainage tube was removed, and the patent was later discharged with a good general condition. A two years' follow-up was done.

## 3 Discussion

Mediastinal teratoma presents in children typically as an anterior mediastinal mass. Other differential diagnoses for an anterior mediastinal mass in children can be thymoma, lymphoma, thymic cyst, lymphangiomas, neurogenic tumors, germ cell tumors and mesenchymal tumors [6].

Mediastinal benign teratomas are well-circumscribed heterogeneous masses that have radiographic characters of multilocular cysts with solid and fatty components in addition to calcifications. These radiographic characters may not be present in every case and only 20–40 % of cases show evidence of calcifications [1]. In our case, calcifications were noted.

Overall, 60 % of patients with mediastinal teratomas are asymptomatic and get diagnosed incidentally during a CXR for other indications [6]. On the other hand, mediastinal tumors cause symptoms that are nonspecific, which are most often associated with tumor growth that compresses the surrounding structures attributed to its mass effect on the mediastinal structures [6], [8]. Symptoms like chest pain, cough, dyspnea, dysphagia or respiratory failure are related to tumor advancement [8]. Our patient presented mainly with upper and lower respiratory symptoms, resulting from the compression by the anterior mediastinal teratoma. Mediastinal mature teratoma is uncommon benign neoplasm, but it could lead to mechanical problems due to its position. Often the treatment could be challenging because the mass could be adherent to major organs and vessels as well to the heart, nerves or lungs, causing surgical difficulties [9]. Teratomas may present with several complications, such as respiratory distress, hemorrhage, pneumothorax or fistula formation into the aorta, the esophagus or bronchus [10].

Determination of serum tumor markers such as αFP and HCG is important in the diagnosis and the follow-up of mediastinal germ cell tumors. This is important as it plays a role in the distinction between immature and malignant teratomas, as patients with benign teratomas do not show elevated tumor markers [11].

CXRs are the initial imaging procedure in diagnosing mediastinal teratomas. Moreover, CT scan is the next step in evaluation for specifying multiples factors such as the nature, location and relationship of the tumor to the other structures. Recently, MRIs have shown a superior diagnostic accuracy over CT scans, particularly in identifying tumor invasion to the capsule and to adjacent structures [12]. However, in our case, delaying the CT scan have led to the delayed diagnosis and prolonging antibiotics use instead of surgical procedure, which emphasizes on the importance of performing a CT scan for recurrent pulmonary symptoms with no response to medical treatment.

The histopathological examination gives the definitive diagnosis. The differential diagnosis includes hydatid cyst, fungal ball, lung abscess and lung parenchymal tumor [13]. Rupture of a pulmonary or mediastinal hydatid cyst can present with symptoms similar to the teratomas such as chest pain, dyspnea and hemoptysis, but can also present with as anaphylactic shock, which is rare. However, the acute onset in case of respiratory distress or anaphylactic shock mostly indicates a ruptured cyst rather than an unruptured cyst or a teratoma [13]. Unfortunately, tumor markers can have a cross-reaction with hydatid antigens [14]. In our case, the diagnosis was thought to be a hydatid cyst due to its epidemiology and its common occurrence in Syria despite having negative hydatid cyst markers.

Complete surgical excision with delicate separation from the surrounding tissue is the treatment of choice for benign mediastinal teratoma [15]. The literature review has described different approaches of mediastinal tumor excision depending on the location and the size [16]. In our case, the excision was done through an anterior lateral dissection through the right chest wall after releasing the mass from its adhesions.

## 4 Conclusion

We present a child who had recurrent pneumonia during an extended period, which were managed by antibiotics, but later became resistance against first-line antibiotics. Delaying the CXR and the CT scan in such as case caused a delayed diagnosis of a mediastinal mature teratoma.

The patient had symptoms and radiological findings of a hydatid cyst, despite having negative serum antibodies; or a lung abscess; both diagnoses were likely due to their etiology and pathology in Syria. However, a teratoma was found to be the cause for the recurrent presentations, which raises the necessity to put benign and malignant tumors in the deferential diagnosis for chest symptoms.

## Abbreviations

WBCs: White blood cells
N: Neutrophils
L: Lymphocytes
Plt: Platelets
Hb: Hemoglobin
CT: Computed tomography
AFP: Alpha-fetoprotein
CRP: C-reactive protein
HCG: Human chorionic gonadotropin
MRI: Magnetic resonance imaging

The case has not been presented at a conference or regional meeting.

## Consent of patient

Patient's parent consent was taken for publishing this case and the images. A copy of the written consent is available for review by the Editor-in-Chief of this journal on request.

## Provenance and peer review

Not commissioned, externally peer-reviewed.

## Ethical approval

Ethical approval was taken from Damascus University's Deanship.

## Funding

This research did not receive any specific grant from funding agencies in the public, commercial, or not-for-profit sectors.

## Guarantor

Ayham Ghareeb.

## Research registration number

This case report is not a first time of reporting, new device or surgical technique. So I would not need a Research Registry Unique identifying number (UIN).

## CRediT authorship contribution statement

**AyG:** Conceptualization, Methodology, Resources, Data curation, Writing - Original draft.
**FA:** Validation, Writing - Review & editing, Visualization.
**AA:** Conceptualization, Methodology, Software, Writing - Original draft, Formal analysis.
**MD:** Validation, Formal analysis.
**AmG:** Investigation, Software, Review & editing.
**HD:** Supervision, Writing - Review & editing, Project administration.

## Declaration of competing interest

We have no conflict of interest to declare.

## Data Availability

All data are available from the corresponding author on reasonable request.
